# Enhancing the UV Emission in ZnO–CNT Hybrid Nanostructures via the Surface Plasmon Resonance of Ag Nanoparticles

**DOI:** 10.3390/nano11020452

**Published:** 2021-02-10

**Authors:** Protima Rauwel, Augustinas Galeckas, Erwan Rauwel

**Affiliations:** 1 Institute of Technology, Estonian University of Life Sciences, 51014 Tartu, Estonia; erwan.rauwel@emu.ee; 2 Department of Physics, University of Oslo, P.O. Box 1048 Blindern, 0316 Oslo, Norway; augustinas.galeckas@fys.uio.no

**Keywords:** ZnO, CNT, nanohybrid, photoluminescence, surface states, Ag nanoparticles, surface plasmon resonance, Burstein–Moss effect

## Abstract

The crystal quality and surface states are two major factors that determine optical properties of ZnO nanoparticles (NPs) synthesized through nonaqueous sol–gel routes, and both are strongly dependent on the growth conditions. In this work, we investigate the influence of the different growth temperatures (240 and 300 °C) on the morphology, structural and crystal properties of ZnO NP. The effects of conjoining ZnO NP with carbon nanotubes (CNT) and the role of surface states in such a hybrid nanostructure are studied by optical emission and absorption spectroscopy. We demonstrate that depending on the synthesis conditions, activation or passivation of certain surface states may occur. Next, silver nanoparticles are incorporated into ZnO–CNT nanostructures to explore the plasmon–exciton coupling effect. The observed enhanced excitonic and suppressed defect-related emissions along with blue-shifted optical band gap suggest an intricate interaction of Burstein–Moss, surface plasmon resonance and surface band-bending effects behind the optical phenomena in hybrid ZnO–CNT–Ag nanocomposites.

## 1. Introduction

ZnO is a well-acknowledged efficient semiconductor phosphor with a wide direct band gap (~3.3 eV), high electron mobility (155 cm^−2^ V^−1^ s^−1^) and large exciton binding energy (60 meV) that ensures effective luminescence at room temperature (RT) [1]. These attractive optoelectronic properties make ZnO a promising multifunctional material for a variety of short-wavelength, light-emitting and detection applications, such as ultraviolet (UV) light emitting diodes (LED), UV lasers and tunable UV photodetectors [2]. In general, ZnO is a highly luminous material exhibiting two distinctive emission signatures in the UV and visible regions of the spectrum, respectively. The former is associated with the near-band-edge (NBE) emission that involves excitonic and shallow-state optical transitions. The other characteristic signature, a broad band in the visible region, originates from a variety of defect states within the band gap and is identified as deep-level emission (DLE) [3]. The luminescent deep states in ZnO are mostly of intrinsic origin, i.e., involving oxygen and zinc vacancies (V_O_, V_Zn_), interstitials (Zn_i_, O_i_) and related complexes. Such point defects may be represented differently at the surface and in the bulk of the crystal; they manifest different charge states and act as donors or acceptors contributing to the visible emission in ZnO [4]. For example, the green emission at 2.3 eV is a surface-related Vo acting as an acceptor state, while the green emission at 2.5 eV is the volume-related Vo acting as a donor state. It is important to note that a visible-light response is of particular interest for various photocatalytic activities [5]. In an attempt to extend the absorption into the visible range, several techniques, such as ion implantation and metal or nonmetal doping, have been investigated [6,7,8]. However, these methods do not appear to be industrially viable in terms of complexity and fabrication costs.

A similar but more cost-effective band gap modification is achievable by introducing Zn and O interstitials and vacancies during the synthesis process itself. In our previous work, we have demonstrated that by using the appropriate precursor it is possible to promote the generation of native defects and consequently modify the absorption and emission properties of ZnO [9]. In that study, nonaqueous sol–gel routes were used for the synthesis of ZnO nanoparticles (NP), and the effects of hydroxyl groups on the physical properties of ZnO were highlighted. Furthermore, the effect of hybridizing ZnO NPs with carbon nanotubes (CNT) further indicated that both the NBE and DLE emissions were enhanced [10]. In all cases, the emission spectra indicated that ZnO had undergone surface modification when linked to CNT, with the newly observed luminescence features linked to the band bending at the ZnO–CNT interface.

Furthermore, significant photoluminescence (PL) enhancement occurs upon hybridizing ZnO NP with CNT. However, in order to maximize photocatalytic activity, these radiative recombination pathways need to be suppressed [11]. Indeed, in photocatalysis, the electron–hole (*e-h*) pairs migrate to the surface of the nanoparticles, where the electrolyte undergoes redox reactions producing O and OH radicals [12] potent enough to degrade organic pollutants and exterminate microbes [13]. In antimicrobial applications in dry media, reactive oxygen species are produced by *e-h* pair separation on the surfaces of the nanoparticles. For both of these activities, the efficient *e-h* pair separation would require restrained radiative recombination pathways manifesting as a quenched PL emission. In addition, UV light irradiation is a complex and expensive method for degrading organics and neutralizing microbes. Therefore, visible-light-activated nanocatalysts, such as ZnO and its hybrids, are being actively pursued as cost-effective alternatives.

An optical band gap increase due to the Burstein–Moss effect has been observed in heavily doped ZnO with a variety of dopants such as Ga [14], Al [15], Gd [16], In [17], Bi [18], Fe [19], Ti [20] and Mg [21]. However, such heavy doping modifies the atomic arrangement and, in turn, the intrinsic properties of ZnO, as it tends to generate secondary phases. Surface plasmon resonance (SPR) induced by noble metal nanoparticles linked to ZnO offers similar advantages in terms of band gap increase and enhanced UV emission without modifying the intrinsic properties of ZnO. In addition, several studies report enhanced photocatalytic and antimicrobial properties along with enhanced UV emission after adding Ag NP to ZnO nanostructures [22]. Prolonging the lifetime of charge carriers by adding other noble metal nanoparticles, such as Au, has also been demonstrated, owing to their SPR effect [23]. However, in terms of cost-effectiveness, Ag appears to be the preferred material when compared to Au. In addition, it is less prone to oxidation as compared to Cu nanoparticles, which also exhibit SPR [24]. In the present study, we address the effects of synthesis conditions and the plasmonic enhancement on the UV emission in ZnO–CNT–Ag nanohybrids. We demonstrate that depending upon the synthesis temperature and the nature of the surface defects in ZnO, the enhancement of PL emission is possible with the addition of Ag NP [25].

## 2. Materials and Methods

### 2.1. Synthesis

The procedure for synthesizing ZnO NP was carried out under air; zinc acetate (3.41 mmol) (99.99%, Aldrich) was added to 20 mL (183 mmol) of benzylamine (≥99.0%, Aldrich). The mixture was poured into a stainless steel autoclave and firmly closed. Thereafter, the autoclave was placed in a furnace at temperatures of 240 or 300 °C for 2 days. The milky suspensions were centrifuged; the precipitates were thoroughly washed with ethanol and dichloromethane and subsequently dried in air at 60 °C. For the ZnO–CNT–Ag nanohybrid synthesis, NANOCYL NC7000 multi-walled carbon nanotubes (MWCNT) with an average diameter of 10 nm and length of 1.5 μm were homogeneously dispersed into the solution of zinc acetate and benzylamine before the solution was transferred into an autoclave for a similar reaction synthesis. Surfactant-free metallic silver nanoparticles of 3 nm diameter were provided by PRO-1 NANOSolutions with >99.9% Ag purity. The supplier guarantees a shelf life of at least 1 year and oxidative stability up to 800 °C indicated by TGA measurements.

### 2.2. Characterization

X-ray diffraction (XRD) patterns were collected using a Panalytical Empyrean diffractometer (Malvern-Panalytical, Netherlands) with a Cu Kα1 radiation source (λ = 0.15406 nm). CHN measurement was conducted using a Leco TruSpec Micro CHNS Analyzer model 630-200-200 (Verder Scientific, Germany) at a temperature of 1075 °C. Carbon, hydrogen and sulfide were measured by infrared absorption, and nitrogen was measured by thermal conductivity. Scanning electron microscopy (SEM) images were recorded on an FEI Quanta 200FEG (FEI, Netherlands). High-resolution transmission electron microscopy (HRTEM) was carried out on a probe-corrected Titan G2 80−200 kV (FEI, Netherlands)and Jeol 200cx (JEOL, Japan), both operating at 200 kV in TEM mode and providing a point-to-point resolution of 2.4 Å. Before the study, the powder was crushed and dissolved in ethanol, and the solution was spread on a carbon-coated grid. Optical absorption properties were derived from the diffuse reflectance measurements performed at room temperature using a ThermoScientific EVO-600 UV–Vis spectrophotometer. Photoluminescence (PL) was investigated at 300 K by employing the 325 nm wavelength of a He–Cd CW laser with an output power of 10 mW as an excitation source. The emission was collected by a microscope and directed to a fiber-optic spectrometer (Ocean Optics, USA) USB4000, spectral resolution 2 nm. The density of the compact powders with and without CNTs was estimated to be approximately the same in all measurements.

## 3. Results and Discussion

Table 1 provides the list of ZnO based samples that were studied in this work. ZnO1 and ZnO2 correspond to ZnO samples synthesized at 240 and 300 °C, respectively. ZnO1–CNT and ZnO2–CNT refer to the two ZnO samples ZnO1 and ZnO2 after conjoining with CNT. ZnO1–CNT–Ag and ZnO2–CNT–Ag are samples containing ZnO1 (synthesized at 240 °C) and ZnO2 (synthesized at 300 °C), combined with CNT and Ag NP. In this study, the terms ZnO–CNT and ZnO–CNT–Ag refer to both ZnO1 and ZnO2 together.

### 3.1. Structure and Morphology

**Figure 1:**
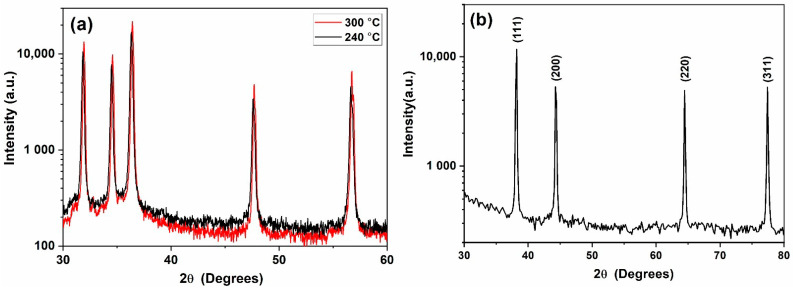
XRD pattern of (**a**) ZnO NP synthesized at 240 and 300 °C and (**b**) free-standing Ag NP.

**Figure 2:**
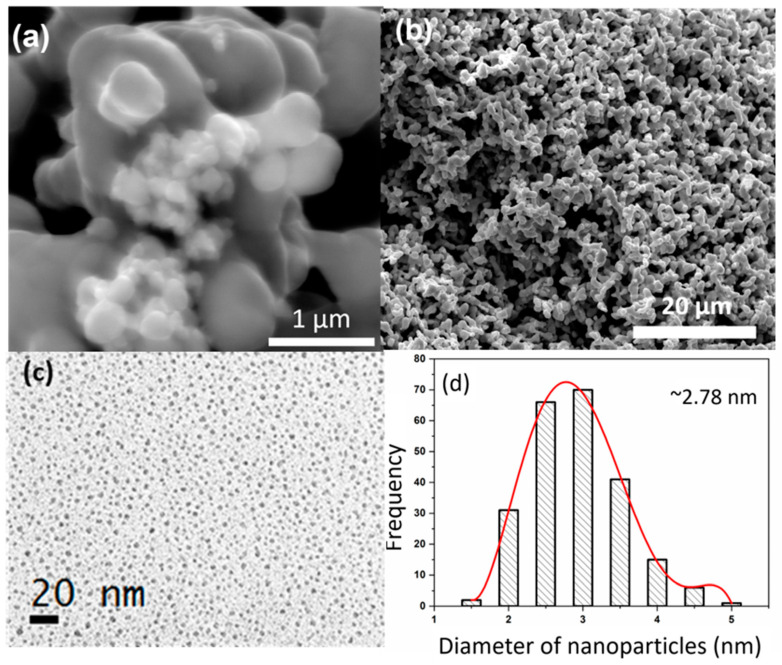
SEM images of Ag NP (**a**,**b**); TEM overview of Ag NP spread on carbon grid (**c**) and their size distribution histogram (**d**).

XRD patterns of the ZnO–CNT synthesized at 240 and 300 °C are provided in Figure 1a. The diffraction patterns correspond to the hexagonal wurtzite-analogous (P6_3_mc) crystal structure (a = 3.25 Å and c = 5.20 Å). The XRD patterns show that ZnO NP are well crystallized without the presence of any secondary phases, with sizes ranging from 80 to 115 nm depending upon the temperature of synthesis [10]. Figure 1b is a typical XRD pattern of the Ag NPs used in this study. It can be clearly seen from the XRD pattern that Ag NP are single-phase and highly crystalline and only exhibit the characteristic diffraction peaks of the Ag face-centered cubic (Fm-3m) structure (JCPDS File No. 87-0720). No secondary phase structure was detected by XRD. The morphology and size distribution of Ag NP were also studied by SEM and TEM. The SEM images in Figure 2a,b illustrates that Ag NP are agglomerated. Nanoparticles smaller than 100 nm can be identified in Figure 2b, which could also be agglomerates of smaller Ag NP. TEM study was subsequently performed to confirm the size of Ag NP. The TEM micrograph presented in Figure 2c illustrates that Ag NP are spherical with an average diameter of 3 nm. The size distribution histogram in Figure 2d provides a mean nanoparticle size of 2.78 nm.

CHN measurements were performed on the Ag nanopowders to measure the quantities of organic elements, if any, on their surfaces. The analysis of 2.0 mg of Ag NP showed that the sample only contains 0.035 wt % carbon, which is most likely due to air contamination.

**Figure 3:**
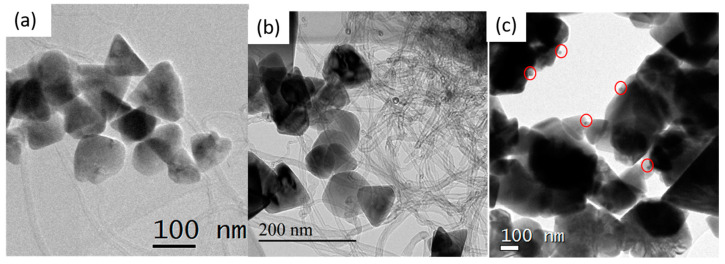
TEM micrographs of (**a**) ZnO1–CNT, (**b**) ZnO2–CNT and (**c**) ZnO1–CNT–Ag. The red circles highlight the Ag NP.

TEM micrographs in Figure 3a,b provide an overview of the ZnO–CNT nanohybrids. The interlaced CNT and ZnO NP form a matrix, thereby ensuring good contact between ZnO and CNTs. Figure 3c presents the hybrid ZnO–CNT–Ag nanocomposite and demonstrates that Ag NP are in direct contact with the ZnO NP. The Ag NPs are circled for clarity in Figure 3c, as at that scale, the smaller Ag NP (2.8 nm in diameter) are barely noticeable against much larger ZnO NP measuring 100–200 nm. The low concentration of only 1 wt % Ag of ZnO–CNT makes their detection challenging.

### 3.2. Optical Properties

**Figure 4:**
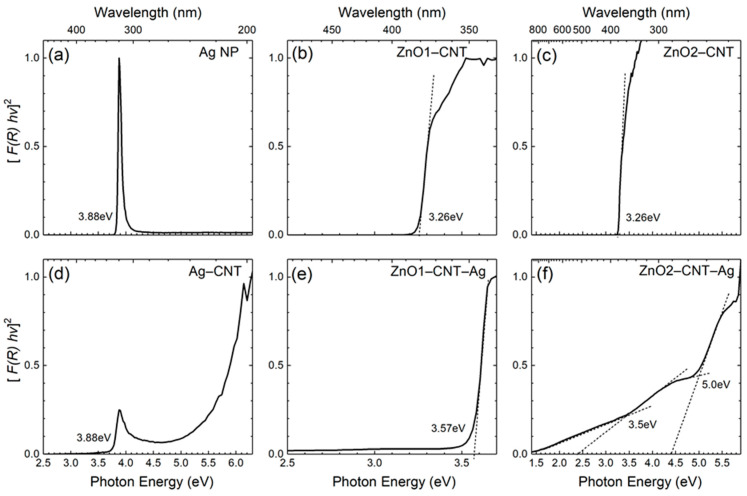
Tauc plots of the (**a**) free-standing Ag NP, (**b**) ZnO1–CNT, (**c**) ZnO2–CNT, (**d**) Ag–CNT, (**e**) ZnO1–CNT–Ag and (**f**) ZnO2–CNT–Ag nanohybrids.

The optical absorption properties of the samples are summarized in Figure 4 with Tauc plots derived from the diffuse reflectance measurements. For pure Ag NP with an average size of 2.8 nm, as follows from the TEM size distribution histogram in Figure 2d, the longitudinal component of the surface plasmon resonance is observed at ~3.88 eV (Figure 4a). This value appears close to the value typically reported in the literature, where an absorption peak at around 4 eV is generally observed. The size reduction and the surfactant or organic ligands on the Ag NP surfaces lead to the redshift of absorption [26]. The Ag NP employed in the present study were surfactant-free (i.e., 0.035 wt % C), and thus the observed minor redshift could only be due to the reduced size of the Ag NP measuring on an average 2.8 nm in diameter. Moreover, once the Ag NP were combined with CNT, the SPR absorption peak slightly redshifted further to 3.7 eV (Figure 4d) due to the realignment of the Fermi levels at the Ag NP and CNT interface, which facilitated electron transfer between Ag NP and CNT [27]. In our previous study, a detailed optical absorption study of the NANOCYL 700 MWCNT revealed their metallic nature [28].

The absorption properties of the ZnO–CNT nanocomposites labelled ZnO1–CNT and ZnO2–CNT are displayed in Figure 4b,c, both exhibiting similar absorption edges at 3.26 eV that match the band gap of ZnO. There are no shoulders extending from the absorption edges into the visible region for either of the two samples. By contrast, for the hybrid nanocomposite containing Ag NP labelled ZnO1–CNT–Ag, the absorption edge at 3.57 eV is observed along with a minor absorption in the range 2.3–3.5 eV (Figure 4e). The band edge energy exceeding the fundamental band gap of ZnO in such a ZnO–CNT–Ag nanohybrid system is indicative of plasmon coupling between the semiconducting ZnO NPs and metallic Ag NP. Indeed, the increased optical band gap of ZnO NP that follows from the blueshifted absorption edge can be explained by the Burstein–Moss (BM) effect typically observed in highly excited or heavily doped semiconductors [29,30]. For a degenerate n-type semiconductor or semimetal, the Fermi level is positioned within the conduction band due to free electrons partially filling this band; i.e., only states above the Fermi level are unoccupied and available for photon capture in a direct optical transition. Consequently, the onset of absorption occurs for photon energies higher than the fundamental band gap, and the magnitude of this BM shift is proportional to the carrier concentration as n^2/3^ [14]. In the present nanohybrids, the carrier concentration increase in ZnO NPs arises from the excess electrons provided by the Ag NP under photoexcitation. As a rough estimation, referring to standard BM effect calculation procedures, the difference between the band gaps of ZnO (E_g(ZnO)_ = 3.26 eV) and ZnO–CNT–Ag (E_g(ZnO–CNT–Ag)_ = 3.57 eV) (cf. Figure 4b,e) creates a BM shift (ΔBM) of ~0.31 eV. This suggests an electron concentration of the order n_e_ ≈ 4 × 10^20^ cm^−3^, and the Fermi level is therefore positioned within the conduction band (CB) at around E_c_ + 0.3 eV. In turn, these parameters provide the necessary clues for further assessment of Schottky or Ohmic barriers and ultimately for building a feasible energy band model behind the optical phenomena of SPR, BM and surface-band-bending (SBB), which is discussed in what follows.

For the hybrid ZnO2–CNT–Ag nanocomposite, two absorption shoulders at ~3.5 and 5 eV can be observed on the Tauc plot in Figure 4f. The absorption edge at 3.5 eV is associated with the BM-shifted optical band gap of ZnO2–CNT–Ag. The absorption edge at 5 eV corresponds to the π plasmon coupling of CNTs and is only visible in this sample [28]. Moreover, a nonzero absorption is also observed throughout the visible region for this sample. The nonzero absorption in the red part of the visible spectrum implies that Zn vacancy-related defects, such as stacking faults, turn optically active under the influence of SPR of Ag NP.

**Figure 5:**
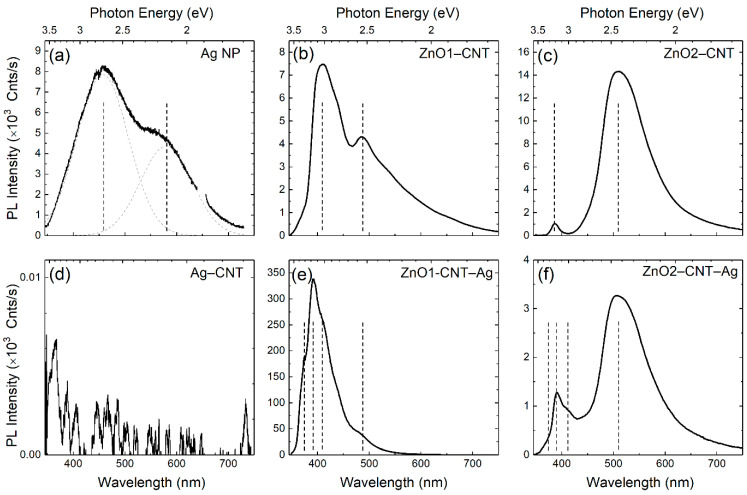
PL emission spectra of (**a**) pure Ag NPs, (**b**) ZnO1–CNT, (**c**) ZnO2–CNT, (**d**) Ag–CNT, (**e**) ZnO1–CNT–Ag and (**f**) ZnO2–CNT–Ag nanohybrids. Vertical dashed markers indicate major emission components discussed in the text.

The optical emission properties of the nanohybrids were assessed by means of room-temperature PL measurements summarized in Figure 5. The employed 325 nm wavelength laser excitation corresponds to 3.81 eV photon energy, which closely matches the plasmonic absorption peak of the Ag NPs at 3.88 eV; i.e., this energy was just sufficient to engender the interband transition of plasmons of the Ag NP. In Figure 5a, the PL emission spectra of the Ag NPs show two main emission peaks centered at 2.7 and 2.12 eV. These emissions with large Stokes shifts of 1.1 and 1.68 eV, respectively, correspond to the electron and hole interband recombination processes in Ag NPs [31]. In the case of Ag clusters with a small average particle size of 2.8 nm, the manifestation of quantum confinement effects is expected [32], which generally consists of the breaking of the band structure into discrete states. The high-energy band at 2.7 eV can therefore be ascribed to the radiative recombination of electrons in the sp-bands with holes in the d-band of agglomerated Ag NP. The low-energy band at 2.1 eV is due to intra-band recombination in the sp-bands of the lowest unoccupied molecular level (LUMO) and highest occupied molecular level (HOMO) gap. However, on combining with CNT and at the same excitation wavelength, total quenching of the luminescence is observed in Figure 5d, suggesting an efficient charge transfer from Ag NP to the CNT. The PL spectra of the ZnO–CNT nanocomposites and hybrid ZnO–CNT–Ag nanostructures are placed alongside for comparison in Figure 5, highlighting the emission changes upon the incorporation of the Ag NPs. For the ZnO–CNT structures, the NBE and DLE emission bands are typical for ZnO nanomaterials [33]. On the other hand, the hybrid ZnO–CNT–Ag nanostructures demonstrate significant extension of emission into the UV region well above the optical band gap of ZnO. The shape of PL emission is typical for the degenerate semiconductor and accounts for both band gap narrowing (BGN) and BM effects. In particular, the low-energy edge of PL spectrum is determined by low-energy transitions from the electron states close to the renormalized gap (i.e., from the bottom of semifilled CB) to acceptor-bound and valence band holes, whereas the high-energy edge is given by the relatively washed out cut-off of the Fermi occupation function at 300 K.

**Figure 6:**
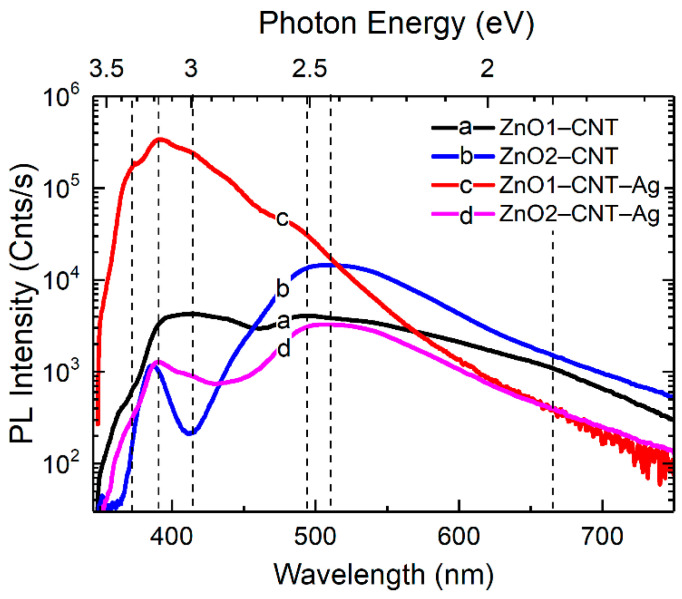
PL spectra at 300 K of (a) ZnO1–CNT, (b) ZnO2–CNT, (c) ZnO1–CNT–Ag and (d) ZnO2–CNT–Ag.

The green emissions at around 2.5 and 2.3 eV represent the bulk or volume and depletion or surface regions of ZnO. The 2.5 eV emission pertains to a transition after a singly ionized oxygen vacancy captures a hole and becomes neutral. In the surface-related Vo emission, a hole is captured by the singly ionized oxygen vacancy, turning it into a doubly ionized oxygen vacancy. This acceptor state then accepts an electron from the conduction band or the depletion region. Nevertheless, for both samples of ZnO–CNT–Ag, there is a loss in the emission activity from the oxygen vacancy related defects when comparing NBE/DLE ratios of ZnO–CNT and ZnO–CNT–Ag (Figure 6). These defect levels of the volume- and surface-related oxygen vacancies lie close to the Fermi level of Ag. The emission in the red region due to transitions from Zn_i_ to O_i_ also shows the same tendency as the green emission. Some of the electrons from the NBE also undergo nonradiative de-excitation and are trapped in defect states such as O vacancies and Zn interstitials. The Ag NPs behave as sinks for these electrons, which are therefore spontaneously transferred to them. On photoexcitation, these electrons are then excited and transferred to higher empty levels of the conduction band, and the entire cyclic process of transfer from defect states of ZnO to the Fermi level of Ag and subsequently to the ZnO conduction band empty states continues. The photoexcitation of the electrons from the Fermi level of Ag can occur by the external excitation of the laser. Another possibility of photoexcitation via the photon emitted by recombination of e-h pairs of the NBE and DLE also exists. These excitations are energetic enough to excite the electrons from the Fermi level of Ag, which are then transferred to unoccupied states of the ZnO conduction band.

**Figure 7:**
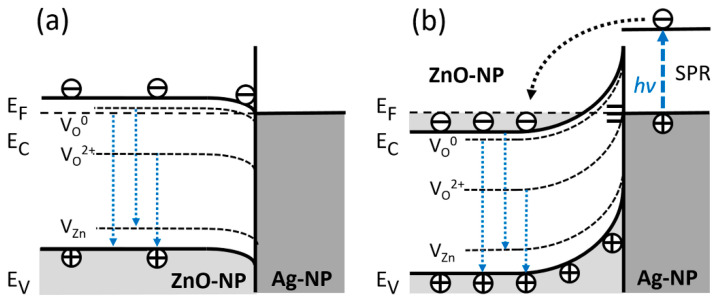
Sketch of optical phenomena in the nanohybrid structures at the interface of ZnO–Ag NP: (**a**) “Ideal” Ohmic contact model, where the interfacial band-bending is downwards because of the larger working function of ZnO than of Ag (~4.7–5.2 eV vs. 4.26 eV, respectively). (**b**) “Realistic” Schottky barrier model with upward band-bending at ZnO NP surfaces induced by interface states of adsorbents, such as hydroxyl groups and oxygen radicals.

Figure 7 presents an outline of a possible model behind the optical phenomena in hybrid ZnO–CNT–Ag nanocomposites. In an ideal case, the downward band-bending model of Figure 7a corresponds to an Ohmic interfacial contact between ZnO and Ag. However, as previously described, absorption measurements (Tauc plots) indicate a blueshifted optical band gap with the Fermi level positioned within the conduction band at around Ec + 0.3 eV. Once an Ag NP comes into contact with a ZnO NP, the Fermi levels of both nanoparticles equalize, meaning an upward band-bending occurs at the surface of the ZnO NP. Furthermore, in a real case scenario, i.e., in air ambient, the upward band-bending in ZnO is also highly expected because of Fermi level pinning to always present surface defects, such as oxygen vacancies (Vo^++^). This kind of upward bending at the ZnO surface implies Schottky barrier (SB) type contact between ZnO and Ag NP along with the presence of a depleted surface region (or space charge region), the width of which depends on several parameters including ZnO NP synthesis conditions as well as, their shape and size. In turn, the surface band-bending affects the intensity of both NBE and DLE, in particular, the green luminescence component associated with the doubly ionized Vo^++^.

In addition, for high interface state densities (Figure 7b), the SB height and band bending become independent of metal work function; i.e., the Fermi level at the surface is pinned by surface states within a narrow energy range in the band gap. In turn, the surface-pinned Fermi level leads to charge transfer that alters the carrier concentration within the surface space charge region, ionizing oxygen vacancy defect states involved in the DLE (green and red luminescence bands). Further, coupling with Ag NP leads to SPR-enhanced electron transfer to ZnO NP up to carrier concentrations causing a degenerate state with the Fermi level positioned within the conduction band, as confirmed by the BM shift in the absorption measurements.

## 4. Conclusions

In this work, we have successfully synthesized ZnO–CNT–Ag nanohybrids. Absorption studies demonstrated that CNTs serve as conducting pathways for the transfer of charges between ZnO and Ag nanoparticles. The plasmon–exciton interactions demonstrated an enhanced UV emission and a suppressed visible light emission in these structures. The widening of the optical band gap of ZnO was attributed to the SPR-induced Burstein–Moss shift [34]. For both ZnO hybrid samples, a nonzero absorption was also observed in the visible region. This suggests that they could be employed in applications such as visible light photodetection, photocatalysis and photovoltaics. Finally, the emission properties under visible light excitation will be assessed in a future work in order to more accurately evaluate the scope of such SPR-enhanced hybrid structures.

## Figures and Tables

**Table 1:** List of samples, their composition and synthesis temperature of ZnO nanoparticles.

Sample	Composition	ZnO Synthesis Temperature (°C)
ZnO1	ZnO	240
ZnO2	ZnO	300
ZnO1–CNT	ZnO and CNT	240
ZnO2–CNT	ZnO and CNT	300
ZnO1–CNT–Ag	ZnO, CNT and Ag	240
ZnO2–CNT–Ag	ZnO, CNT and Ag	300
